# Response Evaluation and Survival Prediction Following PD‐1 Inhibitor in Patients With Advanced Hepatocellular Carcinoma: Comparison of the RECIST 1.1, iRECIST, and mRECIST Criteria

**DOI:** 10.3389/fonc.2021.764189

**Published:** 2021-12-09

**Authors:** Meng Zhou, Chunhui Zhang, Jianhua Nie, Yajuan Sun, Ye Xu, Fangfang Wu, Yuhong Huang, Shun Li, Yuan Wang, Yang Zhou, Tongsen Zheng

**Affiliations:** ^1^ Department of Gastrointestinal Medical Oncology, Harbin Medical University Cancer Hospital, Harbin, China; ^2^ Department of Radiology, Harbin Medical University Cancer Hospital, Harbin, China; ^3^ Department of Phase 1 Trials Center, Harbin Medical University Cancer Hospital, Harbin, China; ^4^ Key Laboratory of Molecular Oncology, Heilongjiang Cancer Institute, Harbin, China

**Keywords:** immunotherapy, PD-1 inhibitor, advanced hepatocellular carcinoma, traditional evaluation criteria, immune-related criteria

## Abstract

**Background:**

Precise evaluation of the efficacy of immunotherapy is critical in the effective management and treatment of advanced hepatocellular carcinoma (HCC). Therefore, the purpose of this study was to compare the response assessments achieved by different criteria and to evaluate the correlation between survival outcome and response assessment in HCC treated with programmed cell death protein 1 (PD-1) inhibitor.

**Methods:**

Fifty patients with advanced HCC treated with first-line PD-1 inhibitor with baseline and follow‐up CT images were analyzed. The patients were categorized into responders and nonresponders according to the criteria.

**Results:**

When the response assessments between RECIST 1.1 and mRECIST were compared, no statistically significant differences were observed. Overall response rate was 16% by RECIST 1.1 and iRECIST and was 24% by mRECIST. According to RECIST 1.1 and mRECIST, overall survival (OS) and progression-free survival (PFS) were not statistically different between the complete response (CR) and partial response (PR) groups and the stable disease (SD) and progressive disease (PD) groups. The OS and PFS were significantly different between responders and nonresponders according to mRECIST. The Cohen’s Kappa for RECIST 1.1, iRECIST, and mRECIST was 0.534, 0.438, and 0.363, respectively.

**Conclusion:**

The mRECIST criteria have a powerful ability to discriminate between responders and nonresponders and demonstrated significantly longer OS and PFS in responders than in nonresponders. However, mRECIST needs to be further improved in order for it to be widely used in the clinical evaluation of immunotherapy in HCC.

## Introduction

Programmed cell death protein 1 (PD-1) expressed on T lymphocytes and activation of the receptors by their ligands (PD-L1) prevents the reaction of T cells to tumor cells and thereby antitumor immunity (1–3). In recent years, additional antibodies targeting the PD-1/PD-L1 have emerged as a promising treatment for inhibiting the progression, relapse, and metastasis of advanced hepatocellular carcinoma (HCC) (4). The initial effect of an PD-1/PD-L1 inhibitor can result in an increase in tumor diameter caused by immune cell infiltration (5). The coexistence of immune cell infiltration and chronic inflammation in HCC might develop an atypical response pattern and patients may initially meet the criteria for progressive disease (PD) but later show stable or reduced tumor burden (6). This can result in failure of the conventional criteria, named Response Evaluation Criteria in Solid Tumors 1.1 (RECIST 1.1) (7), to capture the atypical patterns of tumor response. To address this issue, modified RECIST (mRECIST) (8, 9) and immunotherapy modified Response Evaluation Criteria in Solid Tumors (iRECIST) (10) were introduced, aiming to assist with discrimination between PD and pseudoprogression (PSPD). mRECIST only measures the arterially enhanced parts of the HCC target lesions. iRECIST is based on RECIST 1.1. The major difference between RECIST 1.1 and iRECIST is that the lesion categorized as PD in RECIST 1.1 requires verification by subsequent examinations to distinguish PD from PSPD. A study assessed the efficacy of nivolumab in patients with advanced HCC who achieved an overall response rate (ORR) of 18% by mRECIST and 14% by RECIST 1.1 following second-line treatment (11). Simultaneously, many studies have shown that the conventional criteria (RECIST 1.1) underestimated the benefits of immunotherapy in solid tumors, which may cause an early discontinuation of immune treatment (12–14). In addition, the ORR by mRECIST was regarded as a dependent predictor of overall survival (OS) in HCC treated with targeted therapy, meaning that responders survive significantly longer than nonresponders (15–19). However, there was no consensus on which response evaluation criteria were superior in immunotherapy of HCC, which led to complexities in patient management in different clinical trials. Additionally, the comparisons between the current standard (RECIST 1.1, iRECIST, and mRECIST) assessments are inadequate in advanced HCC. The evidence regarding the association of OS and response assessment by RECIST 1.1, iRECIST, and mRECIST in HCC treated with PD-1 inhibitor is scarce, especially in real-world settings.

The purpose of this study was to compare the response assessments using RECIST 1.1, mRECIST, and iRECIST and to evaluate the correlation between survival outcome and response assessment achieved by these criteria in HCC treated with PD-1 inhibitor.

## Materials and Methods

### Patients

**Figure 1:**
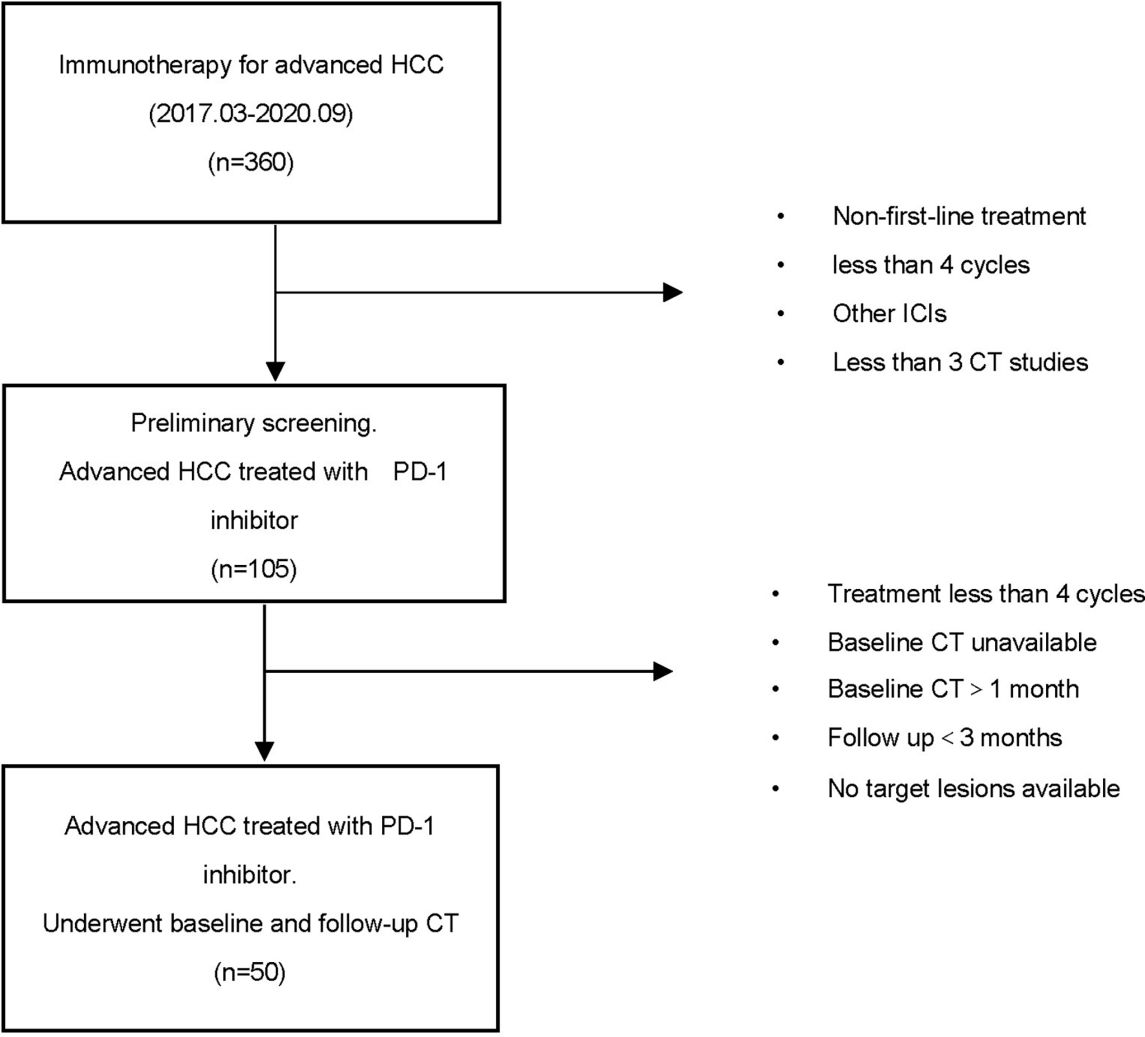
Flow chart shows study population and inclusion criteria.

Patients with advanced HCC receiving PD-1 inhibitor were retrospectively enrolled at the Harbin Medical University Cancer Hospital, China, from March 2017 to September 2020. Eligibility for this research included a diagnosis of HCC and the dosing and treatment duration of PD-1 inhibitor decided according to standard guidelines and treating physicians’ judgment. Patients should also have undergone baseline computed tomography (CT) scans within 1 month before immune treatment and a follow‐up CT scan within 3 months of the last dose of immunotherapy. The main exclusion criteria included the following: patients previously treated with an anti-PD-1, anti-PD-L1, anti-CTLA-4, or other agents targeting T-cell costimulation or immune checkpoint pathways; patients treated with other immunosuppressive medication within 2 weeks before starting PD-1 inhibitor treatment; patients with inadequate radiological evaluation (without an initial CT scan before/after immunotherapy or poor image quality); patients with no target lesions for analysis; and incomplete patient data caused by early death or lost to follow-up (**Figure 1**).

Baseline data at starting PD-1 inhibitor treatment included age, gender, hepatitis B virus (HBV), tumor markers [alpha fetoprotein (AFP)], and types of PD-1 (**Table 1**). This study was approved by the Ethics Review Board of Harbin Medical University. The requirement for informed consent from the patients was waived due to the retrospective design of this study, and patient information was protected.

**Table 1:** General characteristics of the patients, immunotherapy, and CT studies.

Characteristic	Level	Value
Number of patients		50
Age (years, median (IQR))		54 (40–70)
Gender (*n* (%))	Female	47 (94)
	Male	3 (6)
ECOG (*n* (%))	0	47 (94)
	1	3 (6)
BCLC stage (*n* (%))	B	16 (32)
	C	34 (68)
AFP (*n* (%))	>400 ng	21 (42)
	≤400 ng	29 (58)
Drinking history (*n* (%))	Yes	12 (24)
	No	38 (76)
Smoking history (*n* (%))	Yes	17 (34)
	No	33 (66)
HBV history (*n* (%))	Yes	33 (66)
	No	17 (34)
Macrovascular invasion (*n* (%))	Yes	8 (16)
	No	42 (84)
Extrahepatic spread status (*n* (%))	Yes	32 (64)
	No	18 (36)
PD-1 inhibitor (*n* (%))	Nivolumab	8 (16)
	Pembrolizumab	12 (24)
	Camrelizumab	10 (20)
	Tislelizumab	18 (36)
	Sintilimab	2 (4)
ICI cycles between 2 CT studies		2–8 (median: 3)
Further systemic treatments (*n* (%))	Yes	37 (74)
	No	13 (26)

HBV, hepatitis B virus; ICI, immune checkpoint inhibitor.

### Image Analysis

All contrast-enhanced dual-phase CT examinations of the chest, abdomen, and pelvis were performed on a 64-section multidetector CT scanner (128-slice, Siemens Medical System, Erlangen, Germany). The scanning parameters were set as follows: slice thickness and reconstruction interval, 1.25 mm; 120 kV, 250–300 mAs; a matrix of 512 × 512; image reconstruction, 1 mm. To obtain contrast-enhanced image data, a nonionic iodinated contrast agent (iodine concentration: 350 mg/ml) was administered intravenously by a contrast injector at a speed of 4 ml/s. The dynamic contrast-enhanced imaging data were obtained after unenhanced abdominal scanning. We obtained the arterial phase, the portal vein phase, and the equilibrium phase at approximately 30–33, 67–70, and 177–180 s, respectively.

A central radiological review of imaging was evaluated by two radiologists (with 10 and 5 years of experience in CT reading, respectively) independently. If the opinions were not uniform, a third radiologist with 15 years of experience blinded to the results assessed by the others, re-evaluated the images, and disagreements were resolved by discussion. All of the radiologists blinded to the outcome of the patients.

### Response Assessment

As per RECIST 1.1, iRECIST, and mRECIST, the radiologists reviewed the imaging to determine target tumor lesions, measure sum of diameters, assess nontarget lesions, and identify/measure new lesions. All of the radiologists blinded to the outcome of the patients, and then, they analyzed the image information according to RECIST 1.1, iRECIST, and mRECIST finally. Comparing the baseline and follow‐up CT images, response to immunotherapy was classified into four categories according to RECIST 1.1 and mRECIST: complete response (CR), partial response (PR), stable disease (SD), and progressive disease (PD). Immunotherapy assessed by iRECIST was classified into immune CR (iCR), immune PR (iPR), immune SD (iSD), immune-unconfirmed PD (IUPD), and immune-confirmed PD (ICPD). A first PD was assessed by RECIST 1.1, followed by any non-PD response (IUPD) by iRECIST. An ICPD was evaluated on follow-up imaging 4–8 weeks after IUPD. The patients were categorized into responders (CR or PR) and nonresponders (SD or PD) according to RECIST 1.1 and mRECIST. The ORR was defined as CR plus PR rates.

The association between the clinical outcome measure (OS, PFS) and response evaluation were analyzed by an experienced gastrointestinal oncology clinician (with 5 years of experience) who specialized in the diagnosis and treatment of HCC. In addition, the clinician blinded to the outcome of the patients. The OS was defined from the baseline date to the date for all-cause mortality. Progression-free survival (PFS) was defined as the time from the start of PD-1 to the progression of tumors (in any aspect) or death (for any reason). The best overall response (BOR) per RECIST and immune BOR were also assessed. They were obtained with all tumor assessments after initiation of PD-1 inhibitor until documented PD according to the respective criteria.

### Statistical Analysis

Normally distributed continuous variables were expressed as mean ± standard deviation (SD). Independent *t*-tests were performed for continuous independent variables. Cohen’s Kappa test was used to assess the intra- and interobservational reliability between the two radiologists. The coefficients between 0.00 and 0.20, indicated slight agreement; 0.21 and 0.40, fair agreement; 0.41 and 0.60, moderate agreement; 0.61 and 0.80, substantial agreement; and 0.81 and 1.00, almost perfect agreement. Survival analysis curves were drawn using the Kaplan-Meier method, and the Gehan-Breslow-Wilcoxon test was used for comparisons. All statistical analyses were performed using SPSS software (version 22.0, IBM). *p* < 0.05 was considered statistically significant in all comparisons.

## Results

### Response Assessment by RECIST 1.1 and iRECIST

**Figure 2:**
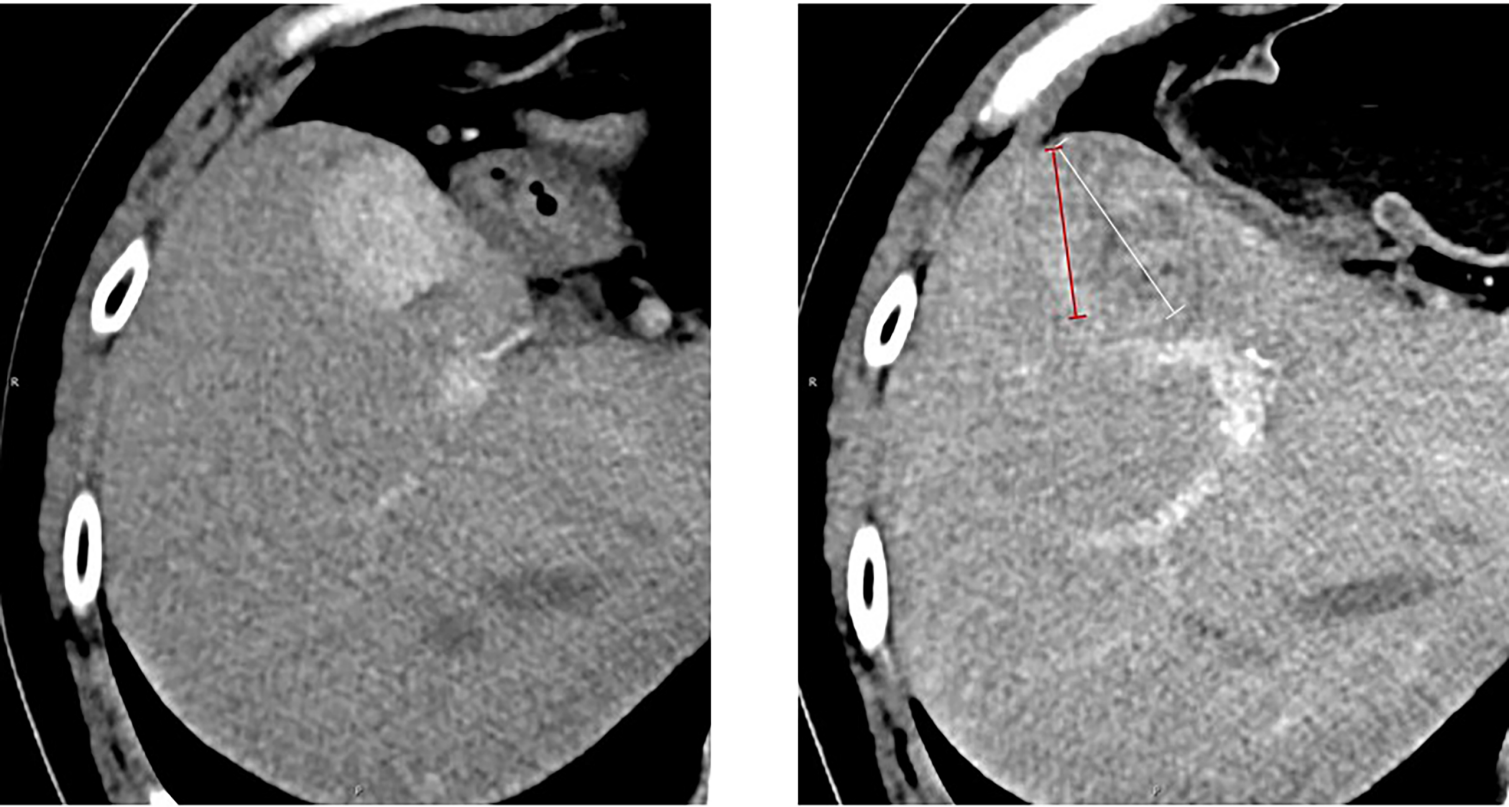
Measurement of the longest tumor diameter in a target hepatic lesion: mRECIST vs. RECIST 1.1. Arterial-phase CT scan obtained after immunotherapy. According to RECIST 1.1, the overall longest diameter of the tumor is captured (white arrow), regardless of the presence of a large area of intratumoral treatment-induced necrosis. In contrast, mRECIST measurement (red arrow) only includes the longest diameter of the viable portion of the tumor, as recognized by contrast enhancement. A 54‐year‐old man who had an arterial-phase CT examination before and after 27 cycles of immunotherapy. The baseline CT size of the target hepatic lesion was 30 mm (left). After immunotherapy, follow‐up CT (right) showed treatment response of previous lesions. The response was assessed as PD according to RECIST 1.1 (47 mm) and SD based on mRECIST (34 mm).

The response assessment of CR/iCR (*n* = 1), PR/iPR (*n* = 7), SD/iSD (*n* = 7), and ORR (16%) by RECIST 1.1 were in common with iRECIST. Discordance between iRECIST and RECIST 1.1 was observed in the assessment of PD. Thirty-five patients (70%) were considered to have PD as per RECIST 1.1 while the disease was considered ICPD (25, 50%) and IUPD (10, 20%) as per iRECIST (**Table 2**). Ten patients were categorized as having IUPD as subsequent scans were unable to be performed during the follow-up period. Each pair was grouped together, leading to two categories: responders (CR and PR) and nonresponders (SD and PD). The response assessment of the two categories was the same between RECIST 1.1 and iRECIST (**Table 3**; **Figure 2**).

**Table 2:** Treatment outcomes per RECIST 1.1, iRECIST, and mRECIST.

*N* = 50	RECIST 1.1	iRECIST	mRECIST
Response No. (%)			
CR/iCR	1 (2)	1 (2)	6 (12)
PR/iPR	7 (14)	7 (14)	6 (12)
SD/iSD	7 (14)	7 (14)	9 (18)
PD/ICPD	35 (70)	25 (50)	29 (58)
IUPD		10 (20)	
ORR	8 (16)	8 (16)	12 (24)

CR, complete response; iCR, immune-complete response; PR, partial response; iPR, immune-partial response; SD, stable disease; iSD, immune-stable disease; PD, progressive disease; iPD, immune-progressive disease; ICPD, immune-confirmed PD; IUPD, immune-unconfirmed PD; ORR, overall response rate.

**Table 3:** The results of univariable analysis comparing RECIST 1.1, iRECIST, and mRECIST criteria.

	CR	PR	SD	PD	*p*-value
RECIST 1.1/iRECIST	1	7	7	35	0.215
mRECIST	6	6	9	29	
*p*-value	0.05	0.766	0.585	0.211	
	Responders		Nonresponders		0.317
RECIST 1.1/iRECIST	8		42		
mRECIST	12		38		

CR, complete response; PR, partial response; SD, stable disease; PD, progressive disease.

**Figure 3:**
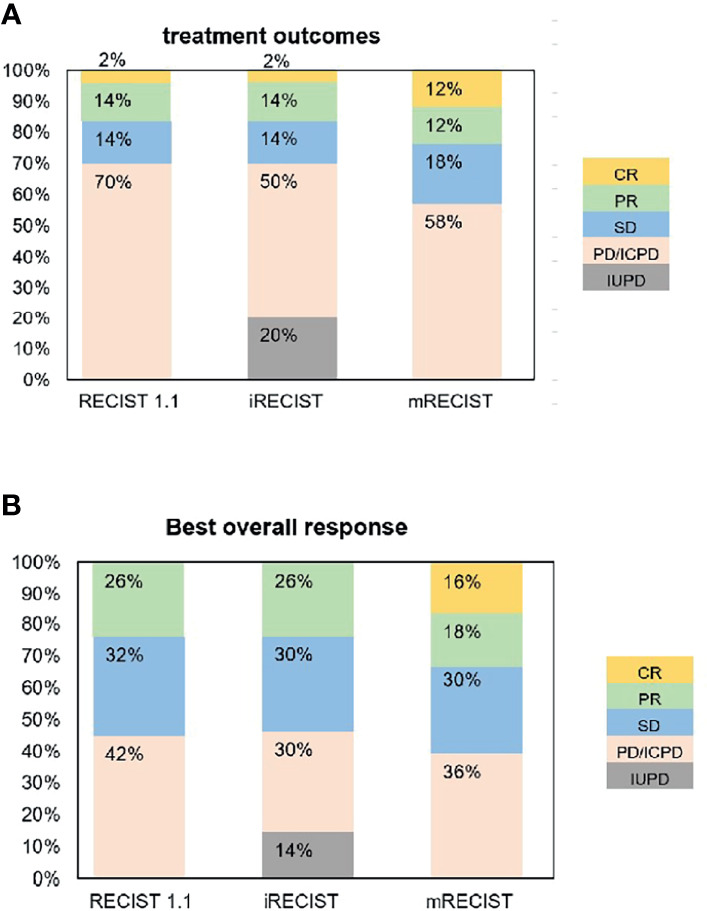
Treatment outcomes **(A)** and best tumor response **(B)** per RECIST 1.1, iRECIST, and mRECIST.

The ORR (16%) by RECIST 1.1 was the same as that by iRECIST (**Table 2**). The BOR by RECIST 1.1 and iRECIST was reported in **Table 4**. The differences in BOR between RECIST 1.1 and iRECIST evaluation were also found in the assessment of SD (*n* = 16, 32% vs. *n* = 15, 30%) and PD (*n* = 21, 42% vs. *n* = 15, 30%). The distribution of response assessment and BOR assessment by RECIST 1.1 and iRECIST is shown in **Figure 3**.

**Table 4:** Best overall response according to RECIST 1.1, iRECIST, and mRECIST criteria.

*N* = 50	RECIST 1.1	iRECIST	mRECIST
Best response No. (%)			
CR/iCR	0 (0)	0 (0)	8 (16)
PR/iPD	13 (26)	13 (26)	9 (18)
SD/iSD	16 (32)	15 (30)	15 (30)
PD/ICPD	21 (42)	15 (30)	18 (36)
IUPD		7 (14)	
CR or PR	13 (26)	13 (26)	17 (34)

(i)CR, (immune-)complete response; PR, (immune-)partial response; SD, (immune-)stable disease; PD, (immune-)progressive disease; ICPD, immune-confirmed PD; IUPD, immune-unconfirmed PD.

### Response Assessment by RECIST 1.1 and mRECIST

Fifteen patients were considered to have an immune response (1 additional CR, 7 additional PR, and 7 additional SD) as per RECIST 1.1 (**Table 2**). Compared with RECIST 1.1, 21 (42%) patients who achieved CR (*n* = 6, 12%), PR (*n* = 6, 12%), and SD (*n* = 9, 18%) were assessed by mRECIST. However, when we compared the response assessment between RECIST 1.1 and mRECIST, no statistically significant differences were found (*p* > 0.05) (**Table 3**).

The ORR was 24% by mRECIST for anti-PD-1 antibody-treated patients, with a slight difference (8%) between RECIST 1.1 (16%) and mRECIST. The BOR by RECIST 1.1 and mRECIST is reported in **Table 4**. The discordance between RECIST 1.1 and mRECIST evaluation was most common for the BOR assessment of CR (*n* = 0, 0% vs. *n* = 8, 16%), PR (*n* = 13, 26% vs. *n* = 9, 18%) and PD (*n* = 21, 42% vs. *n* = 18, 36%). The distribution of response assessment and BOR assessment by RECIST 1.1 and mRECIST is shown in **Figure 3**.

### Correlation of Response Categories With OS

**Figure 4:**
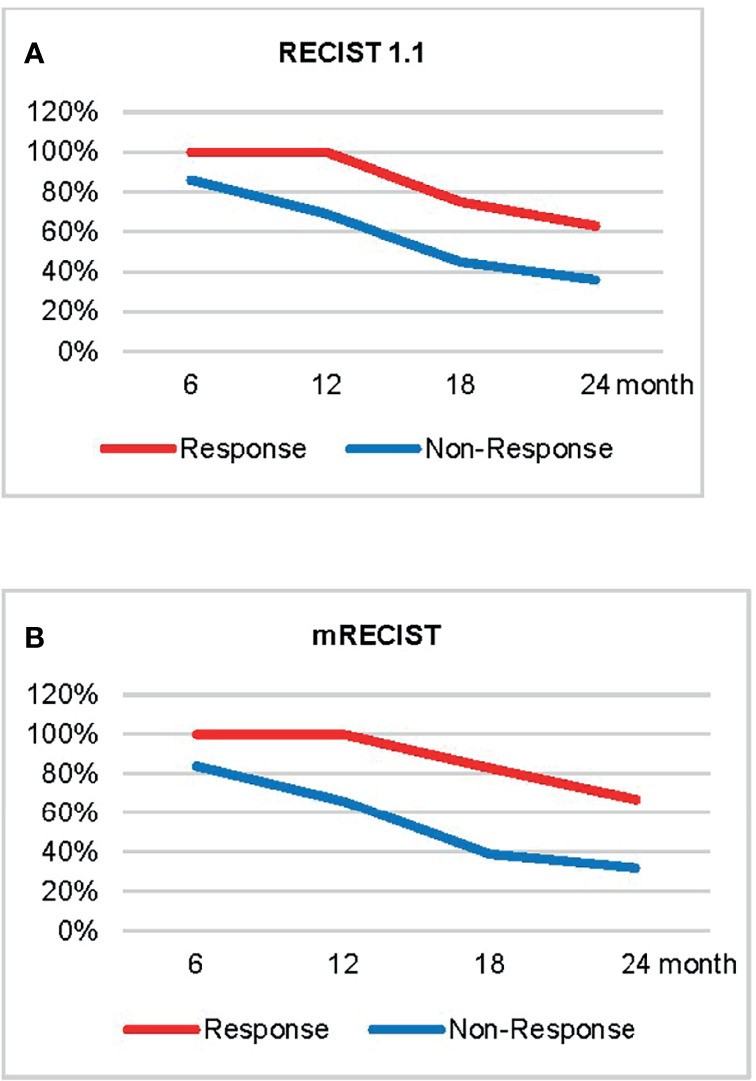
The overall survival rate in different response groups according to RECIST 1.1 **(A)** and mRECIST **(B)**.

The mean OS of the study population was 20.9 ± 12.5 months (range: 2 to 45 months) based on follow-up data. The 6-, 12-, 18-, and 24-month OS rates were 88%, 74%, 50%, and 40%, respectively. **Figure 4** demonstrates the OS rates in the response and nonresponse groups according to RECIST 1.1 and mRECIST.

**Figure 5:**
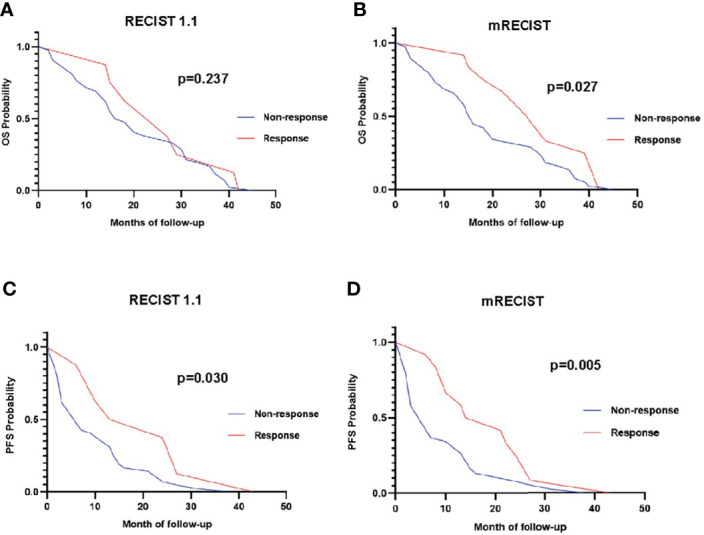
Overall survival curve of responders (red line) versus nonresponders (blue line) using RECIST 1.1 **(A)** and mRECIST **(B)**. Progression-free survival curve of responders (red line) versus nonresponders (blue line) using RECIST 1.1 **(C)** and mRECIST **(D)**.

The OS of anti-PD-1 antibody-treated patients defined by RECIST 1.1 and iRECIST was the same. According to RECIST 1.1 and mRECIST, the OS was not statistically different between the CR and PR groups (*p* > 0.05), and between the SD and PD groups (*p* > 0.05) (**Table 5**). According to RECIST 1.1, the mean OS was 25.5 ± 11.1 months among responders and 20.7 ± 13.0 months among nonresponders (*p* =0.458) (**Table 5**). According to mRECIST, the mean OS was 28.8 ± 10.2 months among responders and 19.0 ± 12.5 months among nonresponders (*p* = 0.018) (**Table 5**). Consequently, the OS was significantly different between responders and nonresponders according to mRECIST (*p* < 0.05). However, there were no significant differences when we compared OS between responders and nonresponders grouped by RECIST 1.1. The differences in OS between responders and nonresponders according to RECIST 1.1 and mRECIST are illustrated in **Figure 5**.

**Table 5:** Compares overall survival (months) between patients with CR and PR and patients with SD and PD and between responders and nonresponders according to different response assessment methods.

	CR	PR	*p*-value	SD	PD	*p*-value
RECIST 1.1/iRECIST	41.0 ± 0	24.6 ± 9.8	0.170	21.1 ± 13.1	20.2 ± 12.9	0.861
mRECIST	32.0 ± 10	24.2 ± 9.4	0.204	19.3 ± 8.2	18.9 ± 13.6	0.915
	Responders			Nonresponders		
RECIST 1.1/iRECIST	25.5 ± 11.1			20.7 ± 13.0		0.458
mRECIST	28.8 ± 10.2			19.0 ± 12.5		0.018

CR, complete response; PR, partial response; SD, stable disease; PD, progressive disease.

### Correlation of Response Categories With PFS

The mean PFS of the study population was 11.5 ± 10.2 months based on follow-up data. The PFS of HCC patients defined by RECIST 1.1 was the same with iRECIST. The PFS were not statistically different between the CR and PR groups (*p* > 0.05) based on RECIST 1.1 and mRECIST, as well as between the SD and PD groups (*p* > 0.05) (**Table 5**). According to RECIST 1.1, the mean PFS was 19.8 ± 12.7 months among responders and 9.9 ± 9.1 months among nonresponders (*p* = 0.011) (**Table 6**). The mean PFS was 18.7 ± 10.8 months among responders and 9.2 ± 9.1 months among nonresponders (*p* = 0.004) (**Table 6**) assessed by mRECIST. Accordingly, the PFS were significantly different between responders and nonresponders according to mRECIST (*p* < 0.05). In addition, the PFS was also statistically different between these two groups according to RECIST 1.1 (*p* < 0.05). The specific differences in PFS between responders and nonresponders according to RECIST 1.1 and mRECIST are illustrated in **Figure 5**.

**Table 6:** Compares PFS (months) between patients with CR and PR and patients with SD and PD and between responders and nonresponders according to different response assessment methods.

	CR	PR	*p*-value	SD	PD	*p*-value
RECIST 1.1/iRECIST	8.0 ± 0	21.4 ± 12.7	0.362	10.3 ± 9.4	8.1 ± 8.0	0.580
mRECIST	22.3 ± 12.7	15.0 ± 7.8	0.256	13.4 ± 8.5	8.1 ± 9.1	0.148
	Responders			Nonresponders		
RECIST 1.1/iRECIST	19.8 ± 12.7			9.9 ± 9.1		0.011
mRECIST	18.7 ± 10.8			9.2 ± 9.1		0.004

CR, complete response; PR, partial response; SD, stable disease; PD, progressive disease.

### Comparison of the Response Assessment of the Two Radiologists

Intrareader variability for all criteria is summarized in **Table 7**. Cohen’s Kappa for RECIST 1.1, iRECIST, and mRECIST was 0.534 (95% CI: 0.305–0.763), 0.438 (95% CI: 0.426–0.450), and 0.363 (95% CI: 0.351–0.375), respectively. Overall, there was moderate agreement for RECIST 1.1 and iRECIST and fair agreement for mRECIST.

**Table 7:** Compare the response assessment of two radiologists.

	Kappa	CI 95%
RECIST 1.1	0.534	0.305–0.763
iRECIST	0.438	0.426–0.450
mRECIST	0.363	0.351–0.375

CI, confidence interval.

## Discussion

Recent immune therapies, including immune checkpoint inhibitors, have shown encouraging clinical results in patients with advanced HCC (20, 21). Programmed death receptor-1 (PD-1)/PD-1 ligand (PD-L1) inhibitors, such as nivolumab and pembrolizumab, have been conditionally approved by the US Food and Drug Administration for second-line treatment of advanced HCC (4, 22). In addition, a number of criteria, including traditional evaluation criteria (RECIST 1.1) and immune-related criteria (iRECIST and mRECIST), have been reported to assess the efficacy of immunotherapy. However, it is unclear which criteria are best for evaluating immune-related response. In addition, the correlations between survival outcome and response assessment achieved by conventional and immunotherapy-modified methods in patients with HCC are also unknown.

This study explored a longer ORR and a greater number of BOR by mRECIST compared with the other criteria. A significant difference between responders and nonresponders assessed by mRECIST (*p* < 0.05) was observed. Our study showed a significantly longer OS and PFS in responders than nonresponders assessed by mRECIST than traditional evaluation criteria (*p* < 0.05).

The tumor assessment by RECIST1.1 is more rigorous and accurate than mRECIST. Patients who have achieved complete tumor remission by mRECIST were responding to immunotherapy actually. Thus, immunotherapy for those patients will be expected to extend the patient’s OS. This means that the mRECIST standard actually prolongs the survival period of some patients who “cannot benefit from immunotherapy”. Furthermore, the intrareader variability of mRECIST was lower than that of the other criteria evaluated in this study and may explain why mRECIST is not widely used in clinical applications and indicates that mRECIST needs to be improved.

Previous studies have shown that conventional criteria underestimated the ORR for immunotherapy by up to 15% (23–26). Simultaneously, it achieved an ORR of 18% by mRECIST and 14% by RECIST 1.1 in second-line treatment of advanced HCC (11). Thus, the conventional criteria (RECIST 1.1) may underestimate the effectiveness of immunotherapy. Our study found that the change in ORR between mRECIST (12 of 50, 24%) and RECIST 1.1 (8 of 50, 16%) was 8% in patients with advanced HCC treated with PD-1 inhibitor. The relatively small sample size in the present research might have affected this result. A recent study has shown that a high ORR to nivolumab in phase II trials was associated with prolonged OS (11). Many studies have indicated that mRECIST criteria in HCC improved the sensitivity to quantify tumor response with targeted therapies (19) and patients who achieved a response on sorafenib had longer survival than nonresponders (27, 28). However, there is little research on the relationship between tumor response and survival in HCC patients treated with immunotherapy. Our study found that OS and PFS of the immunotherapy response group were significantly longer than those in the nonresponse group, which was classified by mRECIST (*p* < 0.05). Hence, our novel findings may suggest that mRECIST is better than RECIST 1.1 and iRECIST in determining immunotherapy response.

The mOS and mPFS of our study population were 20.9 ± 12.5 months and 11.5 ± 10.2 months based on follow-up data. The OS and PFS in our research were longer than previous studies for an immunotherapy monotherapy (5, 29). According to the Child-Pugh and ECOG scores of the patients included in our study, it showed that the patients were in good physical condition. The patients we included had fewer number that occurred macrovascular invasion (16% vs.32%) and less number of extrahepatic metastases (64% vs.71%) compared with CheckMate 040 study. In addition, most patients received further systemic treatments after anti-PD-1 discontinuation. These factors may lead to differences in patient survival analysis between our studies and other clinical studies.

This study attempted to demonstrate the intrareader variability of mRECIST in immunotherapy of HCC for the first time. The concordance rate according to Cohen k was rather low compared with other papers (30). We suggest that the reason for the fair intrareader variability may be caused by the mRECIST measurement method which included measurement of the longest viable tumor diameter for the assessment of response. It is challenging for doctors to accurately measure the longest diameter of the viable tumor in lesions showing partial internal necrosis. Soft tissue resolution of CT is lower than MRI, leading to comparatively low disease detection and measurement sensitivity. Comparatively low disease detection and measurement sensitivity of CT could lead to deviation when determining tumor boundary and measuring tumor diameter. Furthermore, there may be a slight difference in the timing of each CT scan. The drugs used by patients in the two studies were different (PD-1 vs. sorafenib). Special therapeutic responses to tumor immunoassay inhibitor treatment, such as pseudo-progression and hyperprogress, etc., made it more difficult for radiologists with different qualifications. Thus, there is an urgent need for a more reasonable mRECIST assessment that provides a reliable method for assessing tumor response in HCC clinical trials. Quantitative imaging parameters related to immune efficacy evaluation are expected to be added to the mRECIST. Hence, our novel findings may indicate a disadvantage of mRECIST and provide new insights into mRECIST.

There were some limitations in this study. On the one hand, it was a retrospective study from a single center, resulting in recruitment bias. On the other hand, due to the limited number of patients treated with PD-1/PD-L1 inhibitor, further large-scale prospective studies are required to validate the results and examine the clinical utility of mRECIST in advanced HCC patients.

In conclusion, the immunotherapy-modified assessment method (mRECIST) has a powerful ability to discriminate between responders and nonresponders and shows significantly longer OS and PFS in responders than in nonresponders. However, it requires further improvements in order for it to be widely used in the clinical evaluation of immunotherapy in HCC.

## Data Availability Statement

The original contributions presented in the study are included in the article/supplementary material. Further inquiries can be directed to the corresponding authors.

## Ethics Statement

The studies involving human participants were reviewed and approved by Harbin Medical University Cancer Hospital. Written informed consent for participation was not required for this study in accordance with the national legislation and the institutional requirements. Written informed consent was not obtained from the individual(s) for the publication of any potentially identifiable images or data included in this article.

## Author Contributions

Conceived and designed the analysis: TZ and YZ. Collected the data: MZ, YS, YX, JN, FW, and YH. Performed the analysis: MZ, YZ, CZ, SL, and YW. Drafting of the manuscript: TZ and MZ. Wrote the paper: MZ, TZ, CZ, and JN.

## Funding

This study was supported by “Tou Yan Action” of Heilongjiang province, the National Natural Scientific Foundation of China (No. 81472322, No. 81872435, No. 81672930, and No. U20A20377); the Provincial Natural Science Foundation Outstanding Youth Project (JQ2019H003); the National Youth Talent Support Program for TZ; the Natural Science Foundation of Heilongjiang Province (LC201437/H1617); the Fok Ying Tung Education Foundation (No. 151037); Scientific Research Project of the Health Planning Committee of Heilongjiang (2017130); the Academician Yu Weihan Outstanding Youth Foundation of Harbin Medical University for TZ; and the HaiYan Funds of Harbin Medical University for YZ (JJZD2020-17).

## Conflict of Interest

The authors declare that the research was conducted in the absence of any commercial or financial relationships that could be construed as a potential conflict of interest.

## Publisher’s Note

All claims expressed in this article are solely those of the authors and do not necessarily represent those of their affiliated organizations, or those of the publisher, the editors and the reviewers. Any product that may be evaluated in this article, or claim that may be made by its manufacturer, is not guaranteed or endorsed by the publisher.
